# Intracellular Exposure Dose-Associated Susceptibility of Steatotic Hepatocytes to Metallic Nanoparticles

**DOI:** 10.3390/ijms222312643

**Published:** 2021-11-23

**Authors:** Xiaoli Zhang, Yongyi Wei, Chengjun Li, Weiyu Wang, Rui Zhang, Jianbo Jia, Bing Yan

**Affiliations:** 1 Key Laboratory for Water Quality and Conservation of the Pearl River Delta, Ministry of Education, Institute of Environmental Research at Greater Bay Area, Guangzhou University, Guangzhou 510006, China; xiaoli_Zhang19@163.com (X.Z.); cli@gzhu.edu.cn (C.L.); wangweiyu2020@126.com (W.W.); zhangrui20201001@163.com (R.Z.); drbingyan@yahoo.com (B.Y.); 2 School of Environmental Science and Engineering, Shandong University, Qingdao 266237, China; weiyy1991@163.com

**Keywords:** susceptible population, nanotoxicity, steatotic hepatocytes, lipid metabolism, intracellular exposure dose

## Abstract

Non-alcoholic fatty liver disease (NAFLD), mainly characterized by the accumulation of excess fat in hepatocytes, is the most prevalent liver disorder afflicting ~25% of adults worldwide. In vivo studies have shown that adult rodents with NAFLD were more sensitive to metallic nanoparticles (MNPs) than healthy MNPs. However, due to the complex interactions between various cell types in a fatty liver, it has become a major challenge to reveal the toxic effects of MNPs to specific types of liver cells such as steatotic hepatocytes. In this study, we reported the susceptibility of steatotic hepatocytes in cytotoxicity and the induction of oxidative stress to direct exposures to MNPs with different components (silver, ZrO_2_, and TiO_2_ NPs) and sizes (20–30 nm and 125 nm) in an oleic acid (OA) -induced steatotic HepG2 (sHepG2) cell model. Furthermore, the inhibitory potential of MNPs against the process of fatty acid oxidation (FAO) were obvious in sHepG2 cells, even at extremely low doses of 2 or 4 μg/mL, which was not observed in non-steatotic HepG2 (nHepG2) cells. Further experiments on the differential cell uptake of MNPs in nHepG2 and sHepG2 cells demonstrated that the susceptibility of steatotic hepatocytes to MNP exposures was in association with the higher cellular accumulation of MNPs. Overall, our study demonstrated that it is necessary and urgent to take the intracellular exposure dose into consideration when assessing the potential toxicity of environmentally exposed MNPs.

## 1. Introduction

The widespread applications of nanomaterials in various areas such as consumer products [1], agriculture [2], biomedicine [3] and environmental remediation [4] result in their higher environmental release and human exposure. Metallic nanoparticles (MNPs) are among the largest classes of engineered nanoparticles (NPs) found in daily life. For example, commercial products containing MNPs account for 70% of the listed entries with identified NPs supplemented [5]. Environmentally exposed MNPs may enter the human body, interact with biomolecules, and perturb various physiological systems [6,7]. Such adverse outcomes in response to MNP exposure may be further aggravated in susceptible populations characterized by underdeveloped protection mechanisms, impaired self-repair ability, and/or compromised immunity [8,9,10]. Therefore, it is urgent to reveal the potential toxic effects of engineered MNPs to various susceptible populations.

The liver is the main target of various xenobiotic substances including MNPs [11]. Individuals with hepatic disorders (e.g., hepatitis [12] and hepatic steatosis [13]) are, thus, suggested to be more sensitive to environmentally exposed MNPs. Non-alcoholic fatty liver disease (NAFLD) is the most prevalent liver disorder characterized by excess accumulation of fat in hepatocytes, afflicting approximately 25% of the global adult population [14,15]. Exposure to MNPs resulted in aggravated liver injuries in animal models of NAFLD, with enhanced hepatic inflammation and hepatocyte steatosis as the major manifestations [13,16,17,18,19]. However, the complex interplay among different kinds of liver cells in animals has become a limitation for exploring the direct toxic effects of MNPs to the specific type of liver cells.

Hepatocytes are the major parenchymal cells of the liver, comprising 70–85% of liver volume and playing critical roles in metabolism, detoxification, protein synthesis, and innate immunity [20]. In vitro studies showed that exposure to MNPs led to oxidative stress, inflammation, and eventually different types of cell death outcomes in various hepatic cell lines [21]. While the different sensitivities of normal liver cells and hepatic cancer cells to MNP exposure have been reported in several recent studies, with cancer cells more sensitive than normal cells [22,23], the susceptibility of hepatocytes with physiological abnormalities, i.e., steatotic hepatocytes, to direct MNP exposure, remains to be elucidated.

The oleic acid (OA)-induced steatotic HepG2 cell model shows morphological similarities to the hepatocytes of patients with hepatic steatosis and, thus, has been applied as an in vitro model of steatosis for revealing pathogenesis and evaluating the effects of medical or dietary interventions of hepatic steatosis [24,25,26]. In the present study, the toxic potential of several MNPs with high environmental exposure risks to human non-steatotic and steatotic hepatocytes was determined. Both composition and particle size are critical factors in determining the cytotoxicity of MNPs [27,28]. While silver nanoparticles (Ag NPs) and titanium dioxide NPs (TiO_2_ NPs) are the top two MNPs that have the most prevalent usage in consumer products [5], zirconium dioxide NPs (ZrO_2_ NPs) have been widely used for the removal of various pollutants from wastewater, environmental water, and even drinking water [29,30]. Thus, four NPs that have distinct compositions and/or particle sizes were selected to reveal the differential toxicity of MNPs to non-steatotic and steatotic hepatocytes.

## 2. Results

### 2.1. Characterization of MNPs

**Figure 1:**
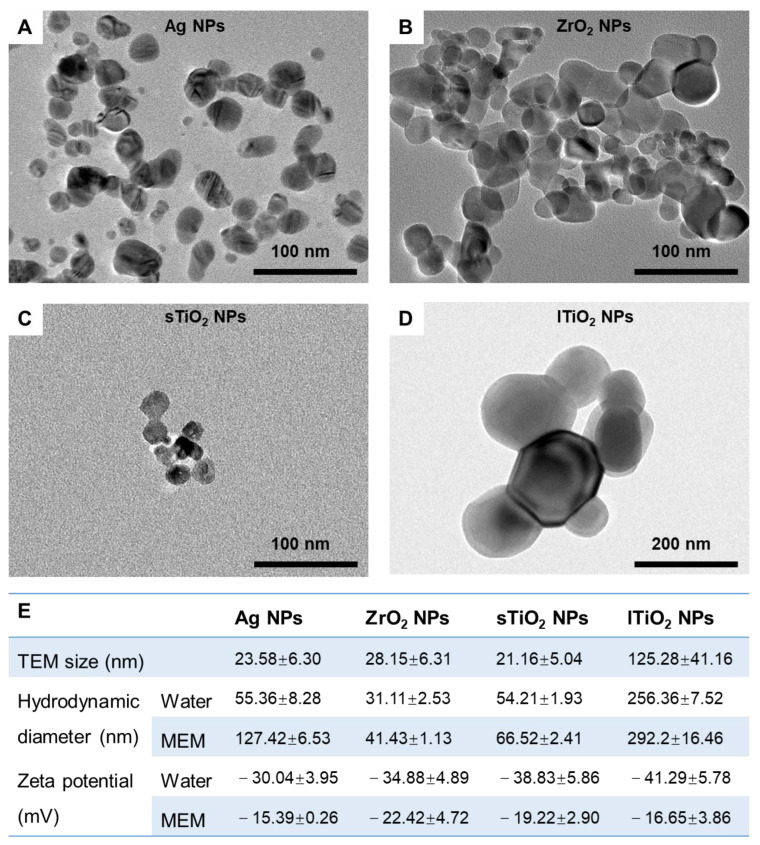
Characterization of MNPs. (**A**–**D**) TEM graphs of Ag NPs (**A**), ZrO_2_ NPs (**B**), small-sized TiO_2_ (sTiO_2_) NPs (**C**), and large-sized TiO_2_ (lTiO_2_) NPs (**D**). (**E**) Summarized characteristics of these four MNPs, including TEM size, hydrodynamic diameter, and zeta potential in DI water and MEM cell culture medium supplemented with 10% fetal bovine serum (FBS). In (**E**), data are shown as means ± s.d., n = 3.

All four MNPs selected were spherical in shape, with transmission electron microscope (TEM) sizes of 23.58 ± 6.30, 28.15 ± 6.31, 21.16 ± 5.04, and 125.28 ± 41.16 nm for Ag NPs, ZrO_2_ NPs, small-sized TiO_2_ (sTiO_2_) NPs, and large-sized TiO_2_ (lTiO_2_) NPs, respectively (Figure 1). The hydrodynamic diameters of these NPs were generally much larger than their TEM sizes, which were further enlarged in minimum essential medium (MEM) cell culture medium supplemented with 10% fetal bovine serum (FBS) due to the adsorption of proteins on the surface (Figure 1E). The formation of protein corona also shifted the surface charges, generally increasing the zeta potential values of these four NPs to similar levels (Figure 1E).

### 2.2. Steatotic Hepatocyte Modeling

**Figure 2:**
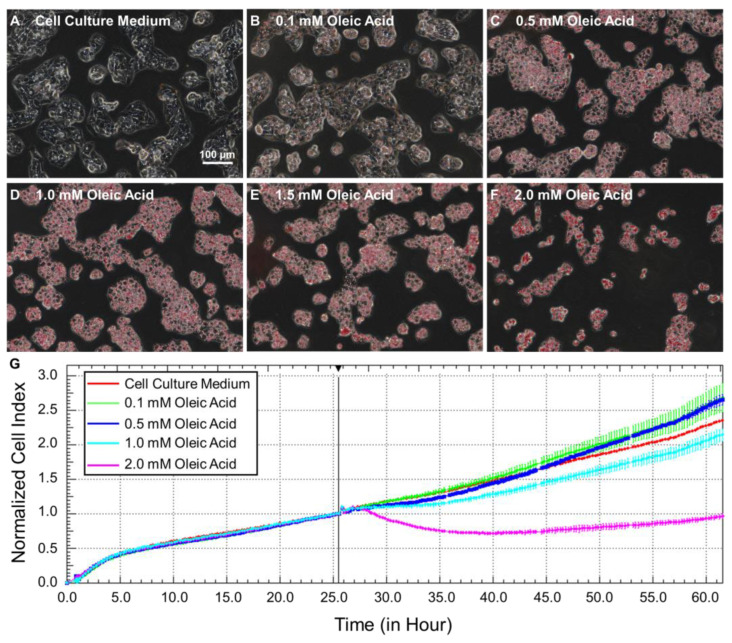
Steatotic hepatocyte modeling. (**A**–**F**) Micrograph of HepG2 cells with oil red O staining after 24 h treatments with OA of various concentrations. (**G**) Real-time growth status of HepG2 cells, as reflected by normalized cell index, in response to OA exposures.

The dose-dependent effects of OA treatments on the accumulation of lipid and growth of HepG2 cells were determined for the selection of a proper dose for steatotic hepatocyte modeling. There was almost an absence of intracellular lipid in cells without the treatment of OA (Figure 2A). OA treatments led to the accumulation of lipid droplets in the cytoplasm of HepG2 cells (Figure 2B–F), at a concentration of ≥0.5 mM. While treatments with 0.1 or 0.5 mM OA showed little effect on the growth of cells, OA exposure at concentrations higher than 1.0 mM resulted in a decreased cell index, suggesting significant alteration of cell growth (Figure 2G). Thus, an OA dose of 0.5 mM, which effectively induced steatosis without affecting the normal growth of HepG2 cells, was selected for steatotic hepatocyte modeling in the following experiments. Following steatotic hepatocyte modeling, the question of whether steatotic hepatocytes were more susceptible to MNP exposure was further investigated.

### 2.3. Higher Cytotoxicity of MNPs to sHepG2 Than to nHepG2

**Figure 3:**
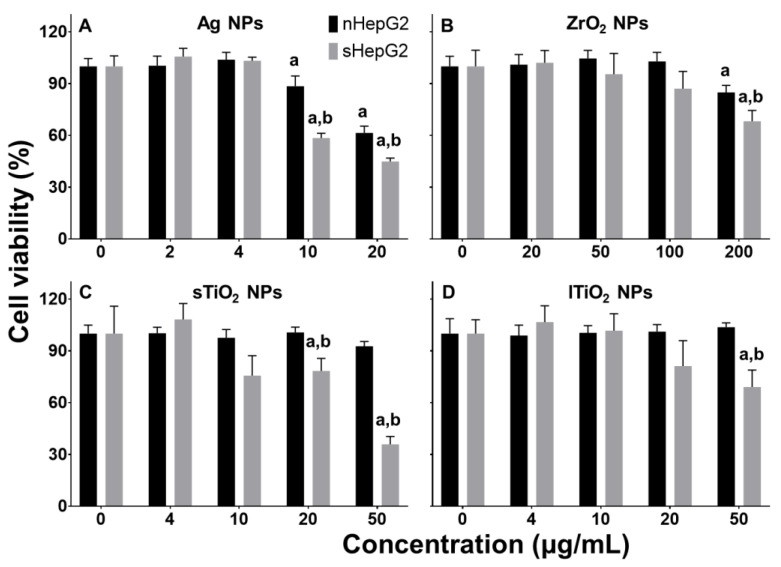
Susceptibility of steatotic hepatocytes to cytotoxicity in response to MNP exposures. (**A**–**D**) Dose-dependent viability of non-steatotic and steatotic HepG2 cells treated with Ag NPs (**A**), ZrO_2_ NPs (**B**), and TiO_2_ of different sizes (**C**,**D**). Data are shown as means ± s.d., n = 5. ^a^ *p* < 0.05, compared with vehicle control. ^b^ *p* < 0.05, compared with viability of nHepG2 cells with the same treatment.

Ag NPs are the most toxic of the four MNPs, causing a decrease in viability of non-steatotic HepG2 (nHepG2) cells at concentrations ≥ 10 μg/mL (Figure 3A). ZrO_2_ NPs caused a slight decrease in viability of nHepG2 cells at concentrations of 200 μg/mL (Figure 3B), and TiO_2_ NPs of any of these two sizes showed little effect on the viability of nHepG2 cells at concentrations of as high as 50 μg/mL (Figure 3C,D). These results indicated the NP composition-dependent cytotoxicity of MNPs, which has been well recognized in previous studies [31,32,33].

In steatotic HepG2 (sHepG2) cells, the toxicity of MNPs is much higher than that in nHepG2 cells. While the viabilities of nHepG2 cells treated with 10 and 20 μg/mL Ag NPs were 88.5% and 61.4%, the same treatments reduced the viabilities of sHepG2 cells to 58.5% and 44.9%, respectively (Figure 3A). In an OA-induced steatotic human hepatocellular carcinoma (HCC) Bel7402 cell model, Ag NP exposure also led to a much higher cytotoxicity than in the non-steatotic (Figure S1). Similar enhanced cytotoxicity to the steatotic hepatocytes, relative to the non-steatotic, was also found for ZrO_2_ NP exposure, as indicated by a much lower cell viability of sHepG2 (68.2%) compared with that of nHepG2 (84.9%) in response to 200 μg/mL ZrO_2_ NPs exposure (Figure 3B). Treatments with 50 μg/mL sTiO_2_ and lTiO_2_ NPs decreased the cell viability to 35.9% and 69.0% only in sHepG2 cells (Figure 3C,D). Despite the similarity in cell type-, dose-, composition- and particle size-dependent cytotoxicity to non-steatotic and steatotic liver cells, our above results demonstrated that the steatotic hepatocytes were more susceptible in cytotoxicity to MNP exposure than the non-steatotic.

**Figure 4:**
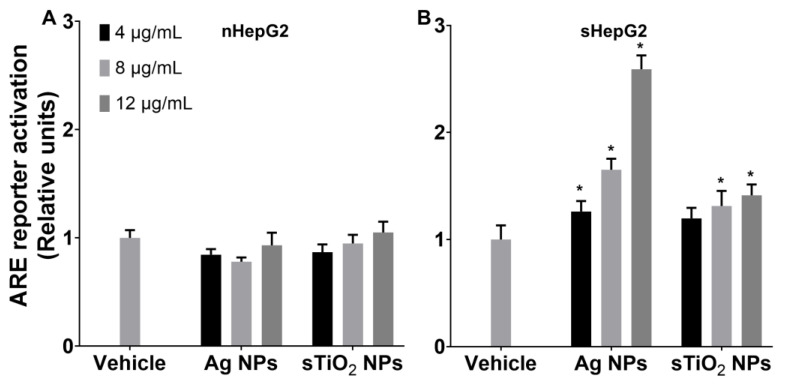
Upregulation of ARE reporter gene only in sHepG2 cells in response to NP exposure. (**A**,**B**) ARE reporter activation in nHepG2 (**A**) and sHepG2 cells (**B**) treated with Ag NPs or sTiO_2_ NPs. Data are shown as means ± s.d., n = 5. * *p* < 0.05, compared with vehicle control.

Using an antioxidant responsive element (ARE) reporter HepG2 cell line (as described in the Materials and Methods), we further investigated the ARE gene expression in nHepG2 and sHepG2 with NP treatments. Ag NPs and sTiO_2_ NPs showed little alteration on the expression of ARE reporter in nHepG2 cells, with the relative units ranging from ~0.8 to ~1.0 (Figure 4A). The ARE reporter expressions in sHepG2 cells exposed to 4, 8, or 12 μg/mL Ag NPs were 1.3-, 1.7-, and 2.6-fold that of vehicle treatment (Figure 4B). Such upregulated expressions of ARE reporter were much lower in sTiO_2_ NP treated sHepG2 cells, with the relative units ranging from 1.2 to 1.4 (Figure 4B).

### 2.4. Alterations of Fatty Acid Oxidation (FAO)-Related Genes in sHepG2 Cells

**Figure 5:**
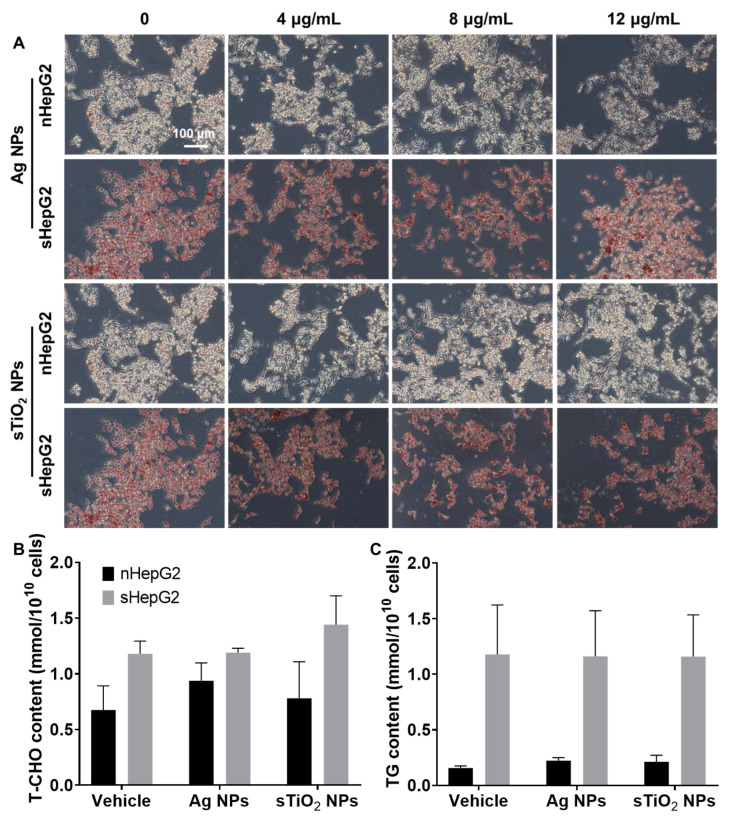
Qualitative and quantitative characterization of intracellular lipids in nHepG2 and sHepG2 cells after various treatments. (**A**) Micrograph of nHepG2 and sHepG2 cells with oil red O staining after 24 h treatments with Ag NPs or sTiO_2_ NPs. (**B**,**C**) Intracellular total cholesterol (T-CHO, (**B**)) and triacylglycerol (TG, (**C**)) contents in nHepG2 and sHepG2 cells treated with 4 μg/mL Ag NPs or sTiO_2_ NPs. In (**B**,**C**), data are shown as means ± s.d., n = 3.

The accumulation of lipid droplets in hepatocytes is one of the most basic pathological characteristics of clinical hepatic steatosis. Here, the alteration of NP exposures on the contents of intracellular lipids and the expression of lipid metabolism-related genes was further investigated. OA treatments led to lipid accumulation in cells and increased intracellular contents of both total cholesterol (T-CHO) and triacylglycerol (TG) (Figure 5). Exposure to MNPs caused few alterations in the intracellular lipids of either nHepG2 or sHepG2 cells (Figure 5).

**Figure 6:**
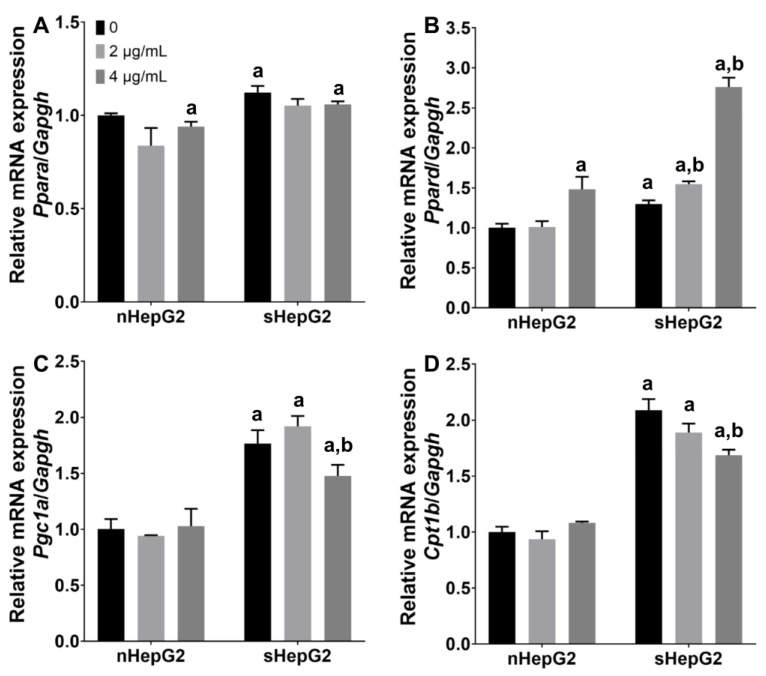
Expressions of FAO-related genes in nHepG2 and sHepG2 cells in response to Ag NP exposure. (**A**–**D**) Relative expressions of *Ppara* (**A**), *Ppard* (**B**), *Pgc1a* (**C**), and *Cpt1b* (**D**) in nHepG2 and sHepG2 cells exposed to 0, 2, and 4 μg/mL Ag NPs for 24 h. Data are shown as means ± s.d., n = 3. ^a^ *p* < 0.05, compared with vehicle control of nHepG2 cells. ^b^ *p* < 0.05, compared with vehicle control of sHepG2 cells.

Despite the negative regulatory effects of MNPs on the intracellular lipids, the expression of several fatty acid metabolism-related genes was further detected in nHepG2 and sHepG2 cells in response to Ag NP exposure at non-lethal doses. The expressions of peroxisome proliferator activated receptor alpha (*Ppara*), peroxisome proliferator activated receptor delta (*Ppard*), peroxisome proliferator activated receptor gamma coactivator 1-alpha (*Pgc1a*), and carnitine palmitoyltransferase-1b (*Cpt1b*) in sHepG2 cells were 1.12-, 1.30-, 1.77-, and 2.09-fold those in nHepG2 (Figure 6), suggesting enhanced FAO in response to OA internalization in sHepG2 cells. While showing little alteration on the expressions of these four FAO-related genes in nHepG2 cells, Ag NPs exposure (4 μg/mL) upregulated the expression of *Ppard* by 112% but downregulated the expression of its coactivator *Pgc1a* and target gene *Cpt1b* by 16% and 20%, respectively, in sHepG2 cells (Figure 6B–D).

### 2.5. Higher Accumulation of MNPs in sHepG2 Than in nHepG2

**Figure 7:**
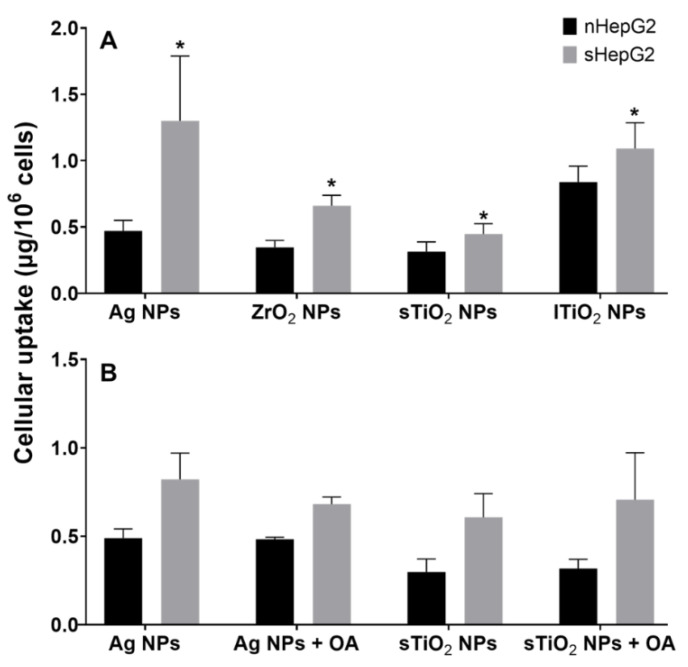
Differential cellular uptake of MNPs by nHepG2 and sHepG2. (**A**) Amounts of NPs in nHepG2 and sHepG2 cells after a 24-h incubation with 4 μg /mL Ag NPs, ZrO_2_ NPs, sTiO_2_ NPs or lTiO_2_ NPs. (**B**) Cell uptake of NPs by nHepG2 and sHepG2 cells following the treatments with Ag NPs or sTiO_2_ NPs for 3 h with/without the supplement of OA. Data were shown as means ± s.d., n = 3. * *p* < 0.05, compared with the amounts of NPs in nHepG2 cells with the same treatment.

At non-lethal dose of 4 μg/mL, MNPs showed higher accumulation in sHepG2 cells in comparison with that in nHepG2 cells. The amounts of Ag NPs, ZrO_2_ NPs, sTiO_2_ NPs, and lTiO_2_ NPs in sHepG2 were 2.8-, 1.9-, 1.4-, and 1.3-fold those in nHepG2 after the cells were incubated with these NPs for 24 h (Figure 7A). Meanwhile, neither the removal of OA from culture medium for sHepG2 cells nor the supplement of OA in medium for nHepG2 cells significantly shifted the accumulation of Ag NPs and sTiO_2_ NPs in these two cells (Figure 7B). These results demonstrate that it is the steatotic status but not the presence of OA that contributes to the higher accumulation of MNPs in sHepG2 than in nHepG2.

## 3. Discussion

Liver toxicity is a pivotal concern for risk assessment of environmentally exposed MNPs, as the liver is the major organ where MNPs are deposited and metabolized. In healthy adult rodents, MNP exposure caused hepatic inflammation, oxidative stress, DNA damage, and hepatocyte apoptosis and/or necrosis [34,35,36]. The above adverse outcomes in response to MNP exposure may be further aggravated in rodent models of NAFLD [13], suggesting the susceptibility of NAFLD individuals to MNPs. Additionally, MNPs aggravated hepatocyte steatosis in NAFLD animal models [16,17]. Hepatic inflammation is a main driver of liver injury, accelerating the transformation of NAFLD from hepatic steatosis to steatohepatitis [37]. Meanwhile, it plays a critical role in regulating the lipid metabolism in hepatocytes. For example, pro-inflammatory cytokine tumor necrosis factor α (TNF-α) disturbed the metabolism of hepatic fatty acid, contributing to the pathogenesis of hepatic steatosis [38,39]. While the induction of hepatic inflammation, as indicated by the increased production of pro-inflammatory cytokines such as TNF-α, interleukin-6 (IL-6), and IL-1β, is one of the most common toxic outcomes of MNPs in both healthy and NAFLD animals [21], the susceptibility of steatotic hepatocytes to direct MNP exposures, especially in regulating the metabolic activity of intracellular lipids, remains to be elucidated. In the present work, the susceptibility of steatotic hepatocytes to four MNPs with different components and particle sizes was first determined in vitro using an OA-induced steatotic HepG2 cell model. Despite the particle size- and component-dependent cytotoxicity of these MNPs to both nHepG2 and sHepG2 cells, MNPs were more cytotoxic to sHepG2 than to nHepG2 (Figure 3), suggesting the susceptibility of steatotic hepatocytes in cytotoxicity to direct MNP exposure.

Induction of oxidative stress is one of the most well-recognized toxic mechanisms of nanosized particles [40]. The Kelch-like ECH-associated protein 1 (Keap1)/NF-E2-related factor 2 (Nrf2)/ARE signaling system is the main antioxidant mechanism against even the lowest level of NP-triggered oxidative stress [41]. The repressor protein, Keap1, acts as a sensor of intracellular oxidative stress, dissociating the transcription factor Nrf2 from the Keap1-Nrf2 complex in response to oxidative stress, and the released Nrf2 then regulates the induction of gene encoding antioxidant proteins via ARE [42,43]. Thus, the ARE reporter has been applied as the most sensitive strategy for measuring the NP-induced oxidative stress response in cells [44]. Using an ARE reporter HepG2 cell line, we compared the induction of oxidative stress in nHepG2 and sHepG2 cells in response to MNP exposure. Treatments with MNPs upregulated the expression of the ARE reporter only in sHepG2 cells (Figure 4), suggesting that the induction of oxidative stress was involved in the susceptibility of steatotic hepatocytes in cytotoxicity to MNP exposure. Previously, the induction of oxidative stress in response to MNP exposure was associated with the inhibition of various detoxification enzymes. One great example is that the inhibition of antioxidant defense systems was shown to be responsible for the gold NP-induced oxidative stress in various human cells [45,46]. Thus, the potential role of the regulatory effects of MNPs on the differential induction of oxidative stress in nHepG2 and sHepG2 cells remains to be elucidated.

The pathogenesis of NAFLD is complex and multifactorial. Peroxisome proliferator-activated receptors (PPARs) are a class of nuclear receptors that have been proven to play critical roles in regulating the metabolism of glucose and lipids, contributing to the development and progression of NAFLD [47]. Among the three PPAR isoforms existing in mammals, both PPARα and PPARδ have high expression in the liver, while PPARγ is highly expressed in adipose tissue [48]. In response to Ag NP exposures, our previous study has shown that the downregulated expression of *Ppard*, and its co-activator *Pgc1a* and target genes contributed to the aggravated hepatic steatosis in overweight mice [17]. However, the question of whether the increased accumulation of lipids in hepatocytes is caused by the internalized Ag NPs in hepatocytes remains to be answered. In the present work, Ag NP exposure showed little effect on the expression of *Ppara*. Meanwhile, treatments with Ag NPs upregulated the expression of *Ppard* but downregulated its co-activator *Pgc1a* and target gene *Cpt1b* (Figure 6). Despite the disagreement of the Ag NP-regulated expression of *Ppard* in vitro and in vivo, the inhibitory potential of Ag NP exposure on the process of FAO is suggested to be the same, as *Ppard* and its co-activator *Pgc1a* together regulate the process of FAO [49]. In contrast, significantly increased accumulation of lipids was not detected in either nHepG2 or sHepG2 cells in response to Ag NP exposure (Figure 5), probably because an exposure time of 24 h was too short.

The intracellular amounts of MNPs increased with the exposure doses, resulting in dose-dependent cytotoxicity of various MNPs, which has been well-recognized in previous studies [50,51,52]. Thus, the MNP-induced cytotoxicity is primarily determined by the intracellular dose of MNPs. In this work, we found that both Ag NPs and sTiO2 NPs had much higher accumulation in sHepG2 cells than in nHepG2 cells (Figure 7a), which was suggested to be responsible for the susceptibility of sHepG2 cells in response to MNP exposure. Even though we excluded the contribution of OA on the differential internalization of MNPs in nHepG2 and sHepG2 cells (Figure 7b), the underlying mechanisms of the enhanced accumulation of MNPs in steatotic hepatocytes remain to be elucidated from how the steatotic status affects the internalization, subcellular localization, and excretion of MNPs in steatotic hepatocytes.

## 4. Materials and Methods

### 4.1. Preparation of MNPs

TiO_2_ NPs of two sizes, Ag NPs and ZrO_2_ NPs, were purchased from Shanghai Macklin Biochemical Co., Ltd. (Shanghai, China), Xuzhou Jiechuang New Material Technology Co., Ltd. (Guangzhou, China), and Yi Fu Co., Ltd. (Shanghai, China), respectively. The morphology and particle size of NPs were analyzed by transmission electron microscope (TEM, JEM-2100F, Jeol, Japan). The hydrodynamic size and the zeta potential of NPs in deionized water or cell culture medium supplemented with 10% fetal bovine serum (FBS, ExCell Bio, Shanghai, China) were measured using the NanoBrook Omni Particle Analyzer (Brookhaven Instruments, New York, NY, USA). The NPs were dispersed in aqueous solutions and sonicated for 30 min before administration.

### 4.2. Cell Culture and Steatotic Hepatocyte Modeling

Human hepatocellular carcinoma HepG2 cells kindly provided by Stem Cell Bank, Chinee Academy of Sciences were cultured in minimum essential medium (MEM, HyClone, UT, USA) containing 10% FBS (ExCell Bio, Shanghai, China), 1% Penicillin-Streptomycin Solution (HyClone, UT, USA), 1% Sodium Pyruvate (Gibco, New York, NY, USA), 1% MEM Non-Essential Amino Acids (Gibco, New York, NY, USA), and 1% GlutaMAXTM-1 (Gibco, New York, NY, USA). The cells were maintained in a 95% humidity and 5% CO_2_ incubator at 37 °C for cultivation. For steatotic hepatocyte modeling, HepG2 cells were seeded in a 12-well plate (60,000 cells per well). After incubation for 24 h, cell culture medium in each well were replaced with fresh culture medium containing 0, 0.1, 0.5, 1.0, 1.5, or 2.0 mM oleic acid (OA) for another 24 h. Cells were washed with PBS three times, stained with oil red O, and analyzed using ECLIPSE Ti2-U Inverted Microscope System (Nikon Instruments Inc., Tokyo, Japan). Real-time growth status of cells in response to OA treatments (0, 0.1, 0.5, 1.0, and 2.0 mM) was determined using an xCELLigence real-time cell analysis system (OLS OMNI Life Science GmbH & Co KG, Bremen, Germany) and reflected as a normalized cell index.

### 4.3. Cell Viability Assay

Cells were seeded in 96-well plates (6000 cells per well). After 24 h incubation, the cells were treated with 0.5 mM OA for 24 h for steatotic hepatocyte modeling and then treated with NPs of various concentrations (with the presence of 0.5 mM OA) for another 24 h. The viability of HepG2 cells was measured using commercialized CellTiter-Lumi^TM^ Luminescent Cell Viability Assay Kit (Beyotime Biotechnology, Shanghai, China) on a Synergy HTX Multi-Mode Reader (BioTek, Winooski, VT, USA).

### 4.4. Antioxidant Responsive Element (ARE) Reporter Gene Assay

ARE firefly luciferase vectors (pGL4.37[luc2P/ARE/Hygro]) were purchased from Promega (Beijing) Biotech Co., Ltd. (Beijing, China). The ARE reporter cell line was prepared by transfecting the pGL4.37[luc2P/ARE/Hygro] vectors into HepG2 cells using Lipofectamine^®^ LTX reagent (Thermo Fisher Scientific China Co., Ltd., Shanghai, China). Resistant colonies were selected using 0.45 mg/mL hygromycin. Stable transfected cells were maintained in MEM supplemented with 10% FBS, 1% Penicillin-Streptomycin Solution, 1% Sodium Pyruvate, 1% MEM Non-Essential Amino Acids, 1% GlutaMAXTM-1 and 0.2 mg/mL hygromycin. To determine the luciferase activity induced by NPs, the ARE reporter cells were treated with OA for 24 h for steatotic hepatocyte modeling and with NPs for another 24 h. Following incubation with Bright-Lumi^TM^ II Firefly Luciferase Assay Kit reagent (Beyotime Biotechnology, Shanghai, China), luciferase activity was measured on a Synergy HTX Multi-Mode Reader (BioTek, Winooski, VT, USA).

### 4.5. Real-Time Quantitative Reverse Transcription PCR (qRT-PCR)

Total RNA of HepG2 cells treated with 4 μg /mL NPs for 24 h was extracted with Trizol reagent (Ambion, Carlsbad, CA, USA). The concentration and quality of RNA was determined using a NanoDrop One spectrophotometer (Thermo Scientific, Waltham, MA, USA). RNA to cDNA were reversely transcribed using PrimeScript^TM^ RT Master Mix (Takara Bio Inc., Shiga, Japan). TB Green^TM^ Premix Ex Taq^TM^ II (Takara Bio Inc., Shiga, Japan) was used to perform quantitative PCR on a LightCycler^®^ 96 Instrument (Roche Molecular Systems, Inc., Pleasanton, CA, USA). The relative expression of genes was quantified using the 2^−ΔΔCT^ method [53]. Primers (Table 1) used in this study were synthesized by Sangon Biotech (Shanghai, China).

### 4.6. Total Cholesterol and Triacylglycerol Levels

Logarithmic growth cells were seeded into 12-well plates. After incubation for 24 h, the normal cell culture medium was replaced by the cell culture medium with/without 0.5 mM OA for cell modeling for another 24 h. Then, the cells were treated with metal-based nanoparticles 24 h. The cells were collected, and the total cholesterol (T-CHO) and triacylglycerol (TG) levels of HepG2 cells after various treatments were determined by T-CHO/TG Assay Kit (Nanjing Jiancheng Bioengineering Institute, Nanjing, China).

### 4.7. Cellular Uptake

HepG2 cells were seeded in 12-well plates at a density of 60,000 cells per well. After a 24 h incubation and a 24 h steatotic hepatocyte modeling, the cells were treated with 4 μg/mL NPs for another 24 h. Cells were then washed three times with PBS, detached by 0.25% trypsin-EDTA solution (Gibco, New York, NY, USA), collected, and counted. The collected cells were dissolved with nitric acid and analyzed using the Inductively Coupled Plasma Mass Spectrometry (ICP-MS, Agilent 7900, Agilent Technologies, Inc., Santa Clara, CA, USA). To explore the effect of OA on the cellular uptake of NPs, cells were exposed to 4 μg/mL Ag NPs or sTiO_2_ NPs for 3 h with/without the supplement of OA, and the intracellular amounts of NPs were determined using the above methods.

## 5. Conclusions

Using an OA-induced steatotic HepG2 cell model, we found that the steatotic hepatocytes were more sensitive to MNP exposures than the non-steatotic in both cytotoxicity and induction of oxidative stress. MNPs showed the potential of perturbing the process of FAO at extremely low doses only in steatotic hepatocytes. The susceptibility of steatotic hepatocytes in response to MNP exposure was in association with the higher cellular accumulation of MNPs, suggesting the necessity and urgency to take the intracellular exposure dose into consideration in the health risk assessment of environmentally exposed MNPs.

## Figures and Tables

**Table 1:** Primers used for qRT-PCR.

Genes	Sequence (5′-3′)	Size (bp)
GAPDH	Forward: GCCTCAAGATCATCAGCAATG Reverse: CCTCCACGATACCAAAGTTGTC	90
PPARA	Forward: CCAGTATTTAGGAAGCTGTCC Reverse: TGAAAGCGTGTCCGTGAT	58
PPARD	Forward: CTACGGTGTTCATGCATGTGAGG Reverse: GCACTTCTGGAAGCGGCAGTA	145
PGC1A	Forward: AATTGAAGAGCGCCGTGT Reverse: AACCATAGCTGTCTCCATC	140
CPT1B	Forward: ACTGCTACAACAGGTGGTT Reverse: TCTGCATTGAGACCCAACTG	76

## Supplementary Materials

The Supplementary Materials are available online at https://www.mdpi.com/article/10.3390/ijms222312643/s1.
