# Supplementary material for: Intracellular Exposure Dose-Associated Susceptibility of Steatotic Hepatocytes to Metallic Nanoparticles
Source: Int J Mol Sci. 2021 Nov 23;22(23):12643. doi: 10.3390/ijms222312643 (PMC8657991; doi:10.3390/ijms222312643)
Supplement: Supplementary file 1 [file ijms-22-12643-s001.zip › ijms-1480478-supplementary.pdf]

Supplementary Materials

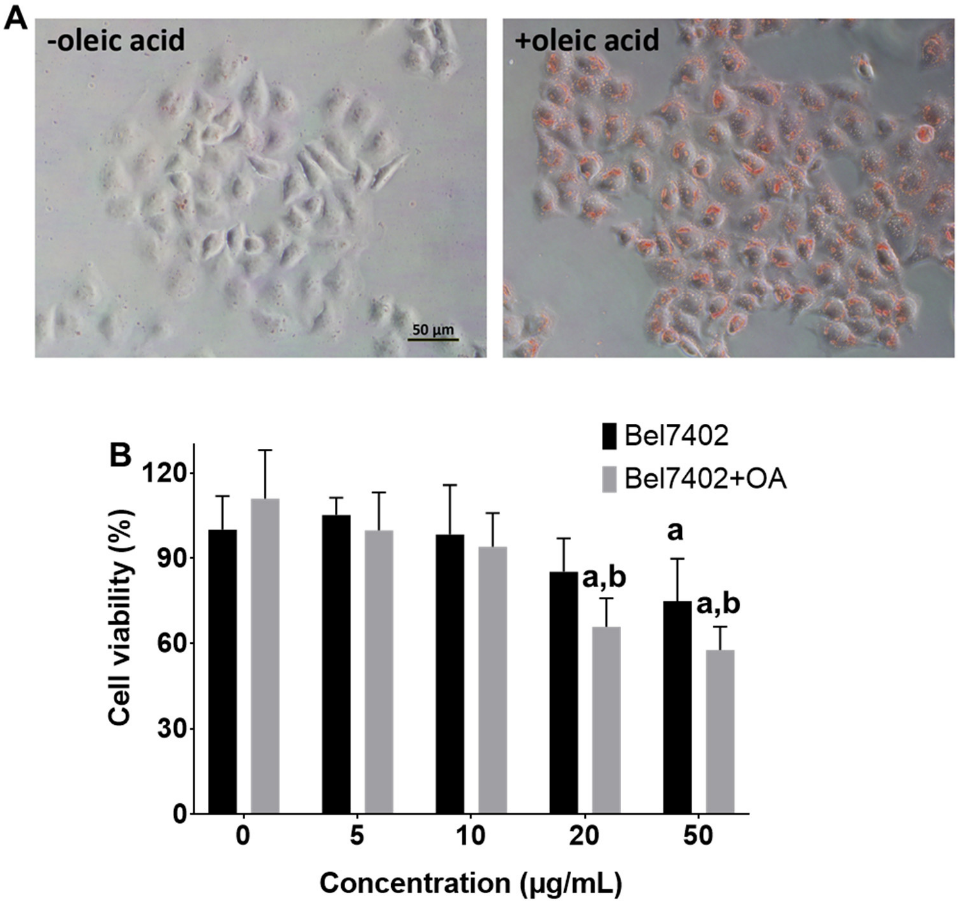

Figure S1. Susceptibility of steatotic Bel7402 cells to cytotoxicity in response to Ag NP exposures. (A) Micrograph of Bel7402 cells with oil red O staining after 24 h treatment with 0.5 mM OA. (B) Dose-dependent viability of non-steatotic and steatotic Bel7402 cells treated with Ag NPs. Data were shown as means ± s.d., n = 5. $^{a}P < 0.05$, compared with vehicle control. $^{b}P < 0.05$, compared with viability of non-steatotic Bel7402 cells with the same treatment.
